# Does Attentional Bias Predict Relapse in Addiction? A Systematic Review of Longitudinal Studies

**DOI:** 10.1002/brb3.70300

**Published:** 2025-02-28

**Authors:** Zehra Su Topbaş, Eda Albayrak Günday, Nuray Şimşek, Emre Usta

**Affiliations:** ^1^ Faculty of Health Sciences, Department of Psychiatry and Mental Health Nursing Yalova University Yalova Türkiye; ^2^ Faculty of Health Sciences, Department of Mental Health and Diseases Nursing Erciyes University Kayseri Türkiye; ^3^ Faculty of Health Sciences, Department of Child Health and Disease Nursing Tokat Gaziosmanpaşa University Tokat Türkiye

**Keywords:** addiction, attentional bias, relapse, substance use disorder

## Abstract

**Purpose:**

The relationship between attentional bias and substance use patterns and cravings in addiction is well documented. However, the connections between attentional bias and relapse remain unclear. This systematic review aims to examine and synthesize longitudinal studies on the relationship between attentional bias and relapse.

**Methods:**

Following PRISMA guidelines, databases including PubMed, Web of Science, EBSCO, and Scopus were searched, yielding 1992 articles. Ultimately, 13 studies were included in this systematic review. Studies were evaluated and coded using a tool developed by the researchers. This review includes studies that explore the associations between attentional bias and relapse related to alcohol and other psychoactive substances.

**Results:**

Findings on the relationship between attentional bias and relapse were inconsistent. However, studies with larger sample sizes demonstrated significant associations between attentional bias and relapse. Generally, the lack of a clear definition of relapse, varied assessment methods, and differences in the implementation of attentional bias tasks contributed to conflicting results.

**Conclusion:**

The findings provide valuable insights for professionals in the field of addiction and researchers focusing on attentional bias. Further research is necessary to elucidate the relationship between attentional bias and relapse, emphasizing the need for clear definitions of relapse and the use of reliable methods to evaluate both relapse and attentional bias. In addition, studies investigating the impact of attentional bias modification on relapse outcomes would be beneficial.

## Graphical abstract

This systematic review comprehensively analyzes longitudinal studies examining the role of AB in predicting relapse. Despite conflicting results among the reviewed studies, significant insights are provided regarding AB's potential predictive role in relapse. The findings underscore the complexity of AB's influence on relapse, shaped by various factors.

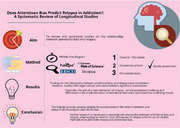

## 1 Introduction

Substance and alcohol addiction is a chronic, relapsing‐remitting mental health disorder characterized by compulsive behaviors, such as drug seeking, that persist despite harmful consequences (NIDA 2020). Patients with addiction exhibit significant variability in symptoms and treatment responses (Milivojevic and Sinha 2018). Consequently, addiction is regarded as a chronic disease marked by periods of remission and relapse, with the primary goal of treatment being the minimization of relapse risk (Ndasauka et al. 2017). However, evidence indicates that relapses following addiction treatment are common (Andersson et al. 2019; Kekic et al. 2022). Therefore, predicting which patients are at high risk of relapse posttreatment and providing specialized interventions for these individuals is crucial.

Previous evidence suggests that substance‐specific attentional bias (AB) in addiction is linked to patterns of craving and substance use. AB refers to the tendency for certain stimuli to capture or hold an individual's attention (Field et al. 2016). In the context of addiction, AB is defined as the unconscious attention to substance‐related cues and is considered one of the most significant cognitive maladaptive changes in chronic drug/alcohol users (Cox et al. 2014). Previous studies present conflicting results regarding the relationship between AB and relapse (Garland et al. 2012; Kennedy et al. 2014; Snelleman et al. 2015). Thus, it is important to examine longitudinal studies on the relationship between AB and relapse through systematic reviews or meta‐analyses.

### 1.1 Background

#### 1.1.1 Attentional Bias and Addiction

AB refers to the automatic and immediate processing of any stimulus by the brain, despite the presence of numerous stimuli from the environment (Field and Cox 2008). This bias toward substance‐related cues is understood as a result of classical conditioning. Classical conditioning involves an organism developing an automatic response to a specific stimulus, such as a substance (Christoforou 2021). Psychoactive substances (e.g., alcohol or drugs) act as unconditioned stimuli, and exposure to these substances elicits unconditioned responses (e.g., pleasure or relaxation) (Field and Cox 2008; Field et al. 2014). Over time, environmental cues linked to the substance (e.g., a particular place, person, or object) become conditioned stimuli. For instance, for an individual who consistently consumes the same brand of alcohol, the logo of the alcohol brand becomes a conditioned stimulus, and the individual may enter a state of reward anticipation. Classical conditioning thus transforms environmental cues associated with addictive substances into conditioned stimuli, while AB enhances sensitivity and attentional orientation toward these cues.

In addition to classical conditioning and AB, the role of reward‐related neurobiological mechanisms in this process is clear. It is known that all addictive substances increase dopamine release in the mesolimbic system and cause feelings of euphoria (Wise and Jordan 2021). The incentive sensitization theory suggests that when an event activates this biological system, the event is noticed, and focused attention on the mental image of this event develops (Robinson and Berridge 1993). Indeed, literature studies provide evidence of increased AB to substance cues in individuals with substance use disorder (SUD) (MacLean et al. 2018; O'Neill et al. 2020). Previous studies also show that AB is associated with the amount and frequency of substance use (Kroon et al. 2023; Weafer and Fillmore 2013). Various experimental paradigms exist for assessing AB (Christiansen et al. 2015). Table 1 provides details about the AB tasks.

**TABLE 1:** Description of attentional bias tasks and measurement methods used in literature.

Task	Measures	Description	Key outcome variable (s)
Stroop Task (Stroop 1935)	Response selection	The Stroop Task consists of three subtasks. Task A (congruent task): Participants are shown one of four words (“blue,” “green,” “red,” or “yellow”) and must read them aloud as quickly as possible. Task B (congruent task): Participants are shown color patches (blue, green, red, or yellow) and must name the color. Task C (incongruent task): Participants are shown the same four words printed in mismatched colors (e.g., the word “blue” printed in yellow ink) and must name the ink color	Interference score: the difference in average response time between incongruent and congruent trials (Task C–Task A). Higher scores reflect greater attentional bias and poorer cognitive control
Dot‐Probe Task (MacLeod et al. 1986)	Response time	Dot‐Probe Task, a computer‐based procedure where a threatening stimulus (e.g., a bottle of alcohol) and a neutral stimulus (e.g., a bottle of water) are simultaneously displayed on the screen. Participants are instructed to focus on a fixation cross in the center of the screen. Subsequently, the threatening and neutral stimuli briefly appear in the right and left corners of the screen. Once the images disappear, a probe is revealed behind one of them. Participants must locate the probe as quickly as possible, typically by pressing specific keyboard keys	The time it takes for participants to detect the dot is measured. Faster responses to dots appearing in the location of the threatening stimulus indicate attentional bias
Visual Search Task (Treisman 1977)	Response time	The visual search task is an experimental paradigm that requires participants to find a specific target stimulus among distractor stimuli. Participants are presented with a series of visual stimuli, which include a target stimulus (e.g., a specific shape or color) and distractor stimuli (similar but different features). Participants attempt to locate the target stimulus among the distractors, determining the presence or absence of the target	The time taken to find the target stimulus is used to assess attentional bias. Shorter response times indicate less attentional bias
Eye‐tracking (Mogg et al. 2003)		With an eye‐tracking device, infrared light is projected onto the participants' eyes and the light is detected by the device. A camera captures the light reflected from the eye and records eye movements. These cameras typically operate at high speeds and can capture hundreds of frames per second. The collected data is analyzed to determine which stimulus the participant looked at and for how long, as well as the direction of eye movements	This analysis includes the points where the eye remains fixed (fixations) and its rapid movements (saccades)
Spatial Orienting Task (SOT) (Derryberry and Reed 1994)	Reward learning	Participants are presented either an upward‐pointing blue arrow (easy target, 75% positive feedback) or a downward‐pointing red arrow (difficult target, 75% negative feedback). They must respond quickly to these cues. After responding, the upward‐pointing blue arrow indicates a quick response (positive feedback), while the downward‐pointing red arrow indicates a slow response (negative feedback). The task consists of four blocks of positive trials and four blocks of negative trials. Participants should aim to score as many points as possible. In positive blocks, quick responses earn 10 points, while slow responses earn none. In negative blocks, slow responses lose 10 points, while quick responses do not lose points	Attentional bias for reward (engagement) refers to faster reaction times to cues of expected gain compared to non‐gain, with lower values indicating a stronger bias. For reward (disengagement), it means slower disengagement from expected gain than from non‐gain, with higher values indicating a stronger bias. Attentional bias for non‐punishment (engagement) involves faster reactions to cues of expected non‐loss compared to loss, with lower values indicating a stronger bias. For non‐punishment (disengagement), it means slower disengagement from expected non‐loss than from loss, with higher values indicating a stronger bias

#### 1.1.2 Attentional Bias and Relapse

While alcohol and substance use determine AB through classical conditioning, repeated substance use progressively disrupts higher‐order cognitive processes. These processes, associated with substance craving and use, are also responsible for relapse. Repeated psychoactive substance use develops a memory specific to the substance (Hayes et al. 2020; Volkow et al. 2011). Even without prolonged exposure to the substance, environmental stimuli can revive memories of the substance, leading to impulsive substance‐taking behavior (Chodkiewicz 2023).

Furthermore, subjective craving that occurs when an individual returns to daily functioning after SUD treatment is known to be a factor in relapse (Schneekloth et al. 2012; Vafaie and Kober 2022). Previous studies have revealed a relationship between AB and substance craving (Bollen et al. 2024; Delonca et al. 2021). Therefore, the relationship between AB and relapse is explained by the occurrence of AB related to the substance together with substance craving. Within the framework of the behavioral approach, this process can be explained by the learning process created by the experience of substance use. Conditional stimuli (such as a lighter, alcohol glass, or syringe) that accompany the person's substance use experience can then elicit conditioned responses such as substance craving, withdrawal symptoms, and so forth (Siegel et al. 2000). In this situation, the individual is likely to pay attention to the conditioned stimuli associated with the reward (Field and Cox 2008). Moreover, the elaborated intrusion theory argues that both internal stimuli (such as withdrawal symptoms and stress) and external stimuli (seeing someone smoking or drinking alcohol) are effective in substance craving. Subjective craving increases when the individual is exposed to external stimuli while under the influence of subjective experiences (Tiffany and Wray 2012).

#### 1.1.3 Aims of the Current Review

This study aims to investigate the relationship between AB and relapse in individuals undergoing treatment for SUD. Given the chronic nature of addiction, characterized by periods of remission and relapse (Milivojevic and Sinha 2018), understanding the cognitive mechanisms contributing to relapse is crucial. Various self‐report tools are available to assess substance craving and relapse cues (Miller and Harris 2000; Rosenberg 2009). However, these measures have limitations, as individuals with substance dependence often exhibit low insight and may report their thoughts and feelings in a biased manner due to social acceptance concerns (Marissen et al. 2005; Steinhoff et al. 2023). Therefore, it is important to evaluate the effectiveness of neurocognitive processes, such as AB, in predicting the development of relapse.

There are systematic reviews and meta‐analyses in the relevant literature addressing this topic. A meta‐analysis conducted by Vafaie and Kober (2022) examined the relationship between drug cues and craving in relation to relapse. However, the study focused not on the direct relationship between AB and drug cues but rather on the relationship between craving resulting from exposure to these cues (regardless of whether AB was measured) and relapse among participants. The systematic review by Christensen et al. (2023) found evidence that neurocognitive predictors play a significant role in the development and persistence of addictive behaviors. Nonetheless, this systematic review did not specifically investigate the relationships between AB related to drug cues and relapse. Thus, it appears that there is no systematic review directly addressing the relationships between AB toward drug cues and relapse.

Theoretical knowledge and empirical studies suggest a potential relationship between AB and the risk of relapse posttreatment, although findings are inconsistent (Garland et al. 2012; Kennedy et al. 2014; Snelleman et al. 2015). This systematic review aims to illuminate the existing scientific knowledge regarding the relationship between AB and relapse, as well as to identify gaps in current research. By determining the role of AB in predicting relapse through previous evidence, this review will discuss the significance of AB in addiction treatment and follow‐up processes, as well as the necessity for potential interventions aimed at reducing AB. The findings of this systematic review are expected to be beneficial for healthcare professionals, educators, and policymakers, providing insights that could enhance treatment strategies and reduce the risk of relapse.

## 2 Methods

This study adhered to the guidelines set forth by the Preferred Reporting Items for Systematic Reviews and Meta‐Analyses (PRISMA) (Page et al. 2021). To mitigate potential bias, the processes of literature search, article selection, and data extraction were independently carried out by the first and second investigators. The data extraction was then reviewed in a session with additional researchers, where a consensus was achieved.

### 2.1 Literature Review

The initial literature review of the systematic review was conducted in November 2023 without any date restrictions. Researchers conducted a second literature review in July 2024. The databases utilized for the search included PubMed, Web of Science, EBSCO, and Scopus. The search was restricted to studies published in English. A combination of various search terms was employed, informed by previous research on AB (Coşkunpınar and Cyders 2013; Field et al. 2009; Vafaie and Kober 2022). The search terms used were: “Addiction OR Substance abuse OR Dependence” AND “Relapse” AND “Attentional bias OR Cognitive bias OR Stroop OR Dot probe OR Dual‐task procedure OR Visual probe task OR Electrophysiological signals OR Reaction time OR Reward learning OR Sign‐tracking OR Spatial cueing task.” In addition to database searches, references from relevant systematic reviews and studies included in the review were manually examined. Supporting Information S2.

### 2.2 Inclusion and Exclusion Criteria

**FIGURE 1:**
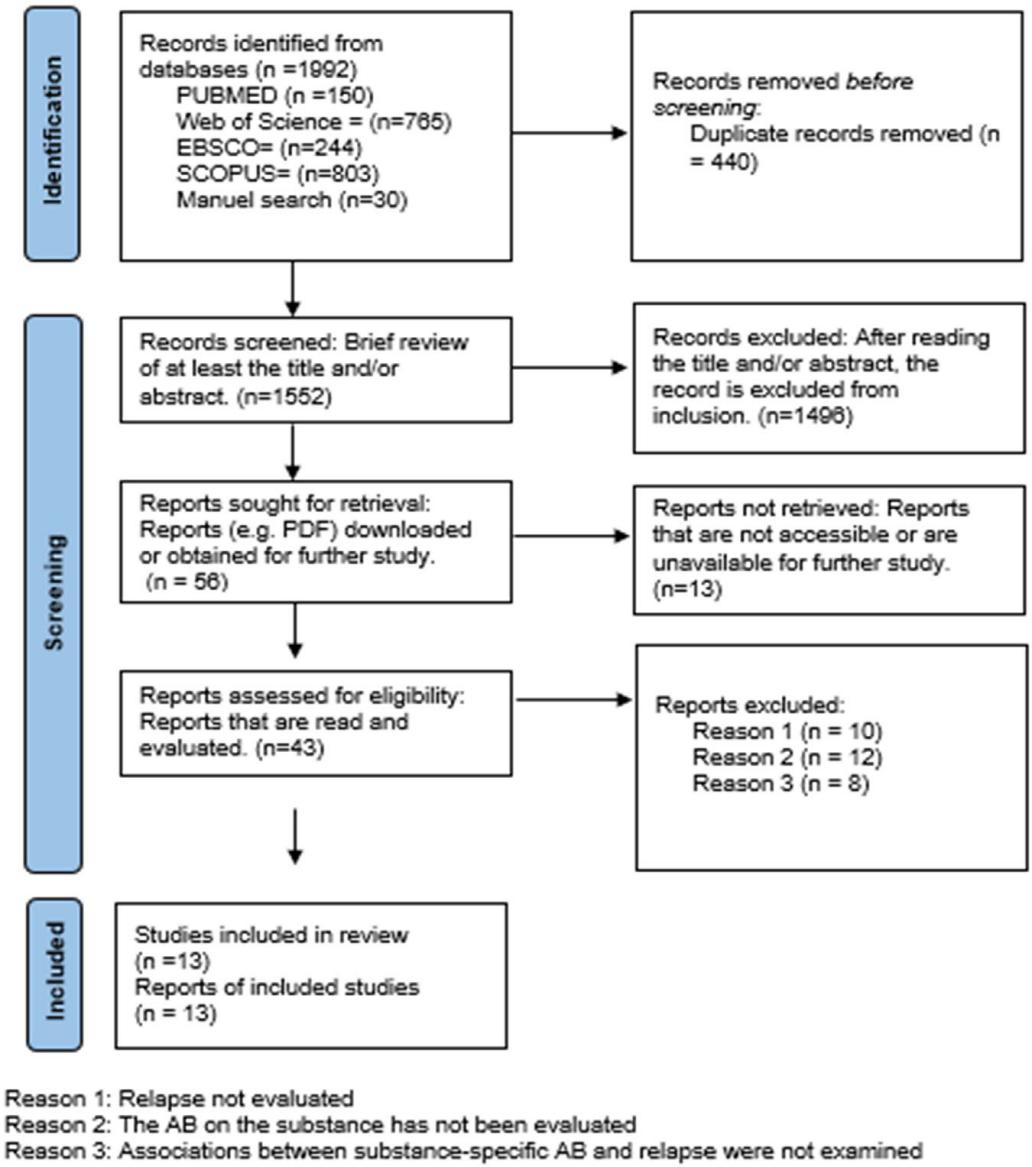
PRISMA flow diagram.

This review exclusively included studies written in English and published in peer‐reviewed journals. The selected studies were longitudinal in nature, focusing on the relationship between AB and relapse. Articles that did not explicitly provide data on relapse follow‐up were excluded. In addition, studies that only addressed subjective craving posttreatment without measuring AB for the specific substance were omitted. Studies measuring AB related to non‐substance cues (such as other rewards) have been excluded. Theses, case series, case reports, book chapters, and conference proceedings were also excluded. Following a comprehensive literature review and quality assessment by the authors, 13 articles were deemed suitable for inclusion in this study (Figure 1).

### 2.3 Evaluating the Quality of Studies

The quality of the articles included in this study was evaluated using the Checklist for Cohort Studies developed by the Joanna Briggs Institute (2017) (Supporting Information S1). This checklist comprises 11 items, each rated as “yes,” “no,” “uncertain,” or “not applicable.” Initially, the first and second researchers independently assessed the quality of the available articles. The results of these assessments were then compared and discussed in a session with all researchers, leading to a consensus (January 2024). Subsequently, five additional potential studies were evaluated for quality, and their appropriateness was discussed in another session with all researchers in August 2024. In instances where the two primary reviewers disagreed on the inclusion of a study, consensus was reached through deliberations involving a meeting of four researchers. Following these discussions, no studies were excluded based on the quality assessment.

### 2.4 Coding and Analysis

A data extraction tool, developed by researchers, was employed to gather the necessary data. This tool encompassed various elements, including the author and publication year of the studies, study location, AB measurement method, sample size and characteristics, number of individuals with and without relapse, timing of relapse assessment posttreatment, relapse assessment method, statistical analysis, and findings. The data extraction process was independently conducted by the first, second, and fourth researchers. Any disagreements were resolved in a session attended by the third researcher. The information of all studies included in the current systematic review is presented in Table 2.

**TABLE 2:** Synthesis of included studies.

Study and location	Sample	AB task	Stimuli in AB task	Evaluation of relapse	Analysis strategy	Outcomes related to relapse	Relationship between attentional bias and relapse
Cox et al. (2002) United Kingdom (UK)	*N* = 14 Male = 11 (78.6%) Female = 3 (21.4%)	Stroop task	– Personalized alcohol vocabulary – Keyboard symbols (******, ∧∧∧∧∧∧, &&&&&&)	– For 3 months after discharge – Self‐reporting	Independent sample *t*‐test	Relapse (−) = 5 Relapse (+) = 9	The score of AB of participants who did not relapse and those who relapsed at Time 1 and Time 2 was not significantly different (*t* < 1.0)
Waters, Shiffman, Bradley, et al. (2003) United States of America (USA)	*N* = 141 (initial sample) *N* = 105 (final sample) Male = 65 (46%) Female = 76 (54%)	Visual probe task	20 cigarette‐related photos 20 neutral photos	– Seven clinical visits were conducted after the quit date, with the last one being at the 3‐month follow‐up. – CO measurements	Cox proportional hazards analysis	Relapse (−) = 105 Relapse (+) = 36	Neither the overall reaction time bias [hazard ratio = 1.00 (95% CIs 0.99–1.01), *p* = 0.66] nor the overall error bias [hazard ratio = 0.99 (95% CIs 0.88–1.13), *p* = 0.94] predicted time to first lapse
Waters, Shiffman, Sayette, et al. (2003) United States of America (USA)	*N *= 158 Male = 76 Female = 82 (52%)	Stroop task	– 11 cigarette‐related words – 11 neutral words	– First week after the Stroop measurement Relapse duration up to the third month (Cox proportional hazards analysis) – Self‐report + CO measurement	Logistic regression Cox proportional hazards analysis	First week Relapse (−) = 73 Relapse (+) = 49	The Stroop measure predicted a 1‐week deprivation. Logistic regression: (OR = 1.50, CI = 0.98, 2.29, *p* = 0.06) Cox survival analysis predicted the initial shift time (hazard ratio [HR] = 1.30, CI = 1.09, 1.54, *p* = 0.003)
Marissen et al. (2006) Netherlands	*N* = 110 (initial sample)/106 (final sample) Male = 98 (89%) Women = 12 (11%)	Stroop task	– 10 words related to heroin – Neutral 10 words	– For 3 months after treatment – Self‐reporting	Logistic regression analysis	*Initial sample* Relapse (−): 84 Relapse (+): 26 *Final sample* Relapse (−): 85 Relapse (+): 21	Stroop effect statistically significantly predicted relapse [OR = 2.23, confidence interval (CI) = 1.06, 4.86, *p* < 0.05]
Carpenter et al. (2006) United States of America (USA)	*N* = 80 Male = 62 (77.5%) Female = 18 (22.5%)	Stroop task	– Words related to cannabis, heroin and cocaine – Neutral words used	– Weekly urine drug test and self‐report – The rate of drug positive urines was calculated (number of positive urines/total number of urines)	Correlation analyses (relationship between positive drug urine rate and Stroop performance scores)	Not clear	Cocaine (*n* = 45): 0.32 (*p* < 0.05) Cannabis (*n* = 25): 0.29 (*p* > 0.05) Heroin (*n* = 10): 0.29 (*p* > 0.05) A significant relationship was found between AB for cocaine and positive urine only in participants whose primary substance of use was cocaine.
Janes et al. (2010) United States of America (USA)	*N* = 19 All participants were women	Stroop task	– Words related to smoking – Neutral words	Weekly CO monitoring and self‐reporting	ANCOVA analysis as (slip, abstinence) × situation (neutral vs. smoking‐related words)	Relapse (−): 11 Relapse (+): 8	There was a significant difference in both reaction time [*F*(1,16) = 7.30, *p* < 0.02] and correct response [*F*(1,16) = 7.98, *p* < 0.015] between participants with and without relapse.
Powell et al. (2010) United Kingdom	*N* = 141 Male = 65 (46.1%) Female = 76 (53.9%)	Stroop Task	– Neutral, appetitive, aversive, and cigarette‐related words	– Approximately 7 days, 30 days, and 3 months after the quit date – Salivary cotinine samples	Logistic regression analysis *χ* ^2^ Tests	– First week Relapse (−): 67 Relapse (+): 74 – First month Relapse (−): 49 Relapse (+): 89 – Third month Relapse (−): 33 Relapse (+): 106	– Only significant on Day 7 (*β* = 0.351, *p* = 0.02) Day 7: (*χ* ^2^ = 6.78, *p* = 0.009) – First month: (*χ* ^2^ = 3.58, *p* = 0.059) – Third month: (*χ* ^2^ = 0.05, *p* = 0.821)
Garland et al. (2012) United States of America (USA)	*N* = 53 (initial sample)/47 (final sample) Male = 42 (79.2%) Female = 11 (20.8%)	Spatial cueing task	– Alcohol stimuli: 13 photos of alcoholic beverages and 7 photos of persons drinking alcohol – Neutral stimulus: 13 photos of kitchen items and 7 photos of persons in kitchen scenes	– Sixth month after treatment – Observation of clinical staff and breathalyzer	Cox proportional hazards model	*Initial sample* Relapse (−): 47 Relapse (+): 6 *Final sample* Relapse (−): 38 Relapse (+): 9	Posttreatment alcohol AB significantly predicted relapse rate and timing (OR = 1.04 [95% CI = 1.00, 1.08], *p* = 0.04
Marhe, Luijten, et al. (2013) Netherlands	*N* = 34 (initial sample)/26 (final sample) Male = 22 (85%) Female = 4 (15%)	Stroop task	– 10 words related to cocaine – 10 neutral words	– Three months after the starting date of work – Self‐report and urine drug test	Progressive regression analysis	Relapse (−): 11 Relapse (+): 15	Stroop effect did not predict relapse (*β* = −0.24, *p* = 0.145)
Marhe, Waters, et al. (2013) Netherlands	*N* = 68 (initial sample) *N* = 64 (final sample) Male = 58 (85.3%) Female = 10 (14.7%)	– ‐Stroop task – ‐Ecological momentary assessment (EMA) Stroop Task	Ecological momentary assessment (EMA) Stroop task – Heroin EMA12 heroin‐related words12 neutral words – Cocaine EMA12 cocaine‐related words12 neutral words	– From the third day to the ninth day after the start of the study (early relapse) – Relapse after the study (late relapse)Self‐report	Logistic regression analysis Linear mixed models (LMM)	– Early relapseRelapse (−): 54Relapse (+): 10 – Late relapseRelapse (−): 29Relapse (+): 25	– LLM results*Early relapse*Significant effect (*F*(1,688) = 5.61, PE = 108.0, SE = 45.6, *p* < 0.025).*Late relapse*The interaction of late relapse status was not significant for EMA (*p* > 0.1). – Logistic regression*Early relapse*Significant effect (PE = 0.0014, SE = 0.00062, Wald = 4.94, *p* < 0.05)*Late relapse*No significant effect
Mitchell et al. (2013) United States of America (USA)	*N* = 15 Male = 6 (40%) Female = 9 (60%)	Stroop Task	Cocaine cues and neutral cues	Self‐report and urine results	Pearson correlation coefficient	Not clear	The Stroop effect was positively correlated with the reported maximum number of consecutive days of cocaine withdrawal during treatment (*r* = 0.78, *p* < 0.05) and negatively correlated with the percentage of positive urine samples (*r* = −0.66, *p* < 0.05)
Kennedy et al. (2014) United States of America (USA)	*N* = 35 All participants were male	Stroop task	– Personalized cocaine words and neutral words (cocStroop task) – Task consisting of congruent stimuli with the same word number and name and incongruent stimuli with different word number and name (cStroop)	– Within 3 months after the start of study – Urine drug test, self‐report and other reports	Logistic regression analysis	Relapse (−): 20 Relapse (+): 15	cocStroop (neutral cocaine) and cStroop (incongruent‐congruent) interventions did not predict relapse (cocStroop model: *p* = 0.115, OR = 1.01, confidence intervals (CI) = 0.99, 1.02 and cStroop model: *p* = 0.250, OR = 1.01, CI = 0.99, 1.02)
Snelleman et al. (2015) Netherlands	*N* = 59 (initial sample)/50 (final sample) Male = 39 (77.8%) Female = 11 (22.2%)	Stroop task	– Words related to alcohol – Neutral words	– One month after the first interview and for 3 months after the second interview – Self‐reporting	Independent sample *t*‐test	*Final sample* Relapse (−): 30 Relapse (+): 20	In Session 1, there was no significant difference in the AB scores of those with and without relapse (*t*(46) = 0.591, *p* = 0.558, Cohen's *d* = 0.17) In Session 2, there was no significant difference in the AB scores of those with and without relapse (*t*(40) = −0.62, *p* = 0.54, Cohen's *d* = 0.07)

## 3 Findings

### 3.1 Characteristics of the Studies

The studies included in this systematic review were conducted between 2002 and 2015. Specifically, two studies were conducted in the United Kingdom (Cox et al. 2002; Powell et al. 2010), four in the Netherlands (Marhe, Luijten, et al. 2013; Marhe, Waters, et al. 2013; Marissen et al. 2006; Snelleman et al. 2015), and seven in the United States (Janes et al. 2010; Carpenter et al. 2006; Garland et al. 2012; Kennedy et al. 2014; Mitchell et al. 2013; Waters, Shiffman, Bradley, et al. 2003; Waters, Shiffman, Sayette, et al. 2003). In all these studies, participants' AB towards the psychoactive substance was initially measured, followed by a longitudinal follow‐up to monitor relapse. The information about these studies is organized under specific headings.

### 3.2 Sample Characteristics

The majority of participants in the studies met the DSM‐IV diagnostic criteria. Only two studies, by Waters, Shiffman, Bradley. et al. (2003) and Waters, Shiffman, Sayette, et al. (2003), included participants who had been smoking for 5 years, averaging 15 cigarettes per day. Three studies focused on individuals diagnosed with alcohol dependence (Cox et al. 2002; Garland et al. 2012; Snelleman et al. 2015), while four studies examined those with tobacco dependence (Janes et al. 2010; Powell et al. 2010; Waters, Shiffman, Bradley, et al. 2003; Waters, Shiffman, Sayette, et al. 2003). In addition, two studies included participants with cocaine dependence (Kennedy et al. 2014; Marhe, Luijten, et al. 2013), and one study investigated heroin dependence (Marissen et al. 2006). Carpenter et al. (2006) conducted a study involving individuals diagnosed with cocaine, cannabis, and heroin addiction. Marhe, Waters, et al. (2013) included both heroin and cocaine users in their research. Across all studies, the total number of participants was 904. Some studies reported participant characteristics based on excluded individuals, while others reported based on the final sample. Of the participants, 579 (64%) were male and 325 (36%) were female. The average age of participants ranged from 21.4 years (Marissen et al. 2006) to 48.7 years (Snelleman et al. 2015).

In the studies, individuals with Axis I disorders (Janes et al. 2010; Carpenter et al. 2006; Kennedy et al. 2014; Marhe, Luijten, et al. 2013; Marhe, Waters, et al. 2013; Mitchell et al. 2013; Powell et al. 2010), Axis II disorders (Snelleman et al. 2015), and color blindness (Marissen et al. 2006; Marhe, Luijten, et al. 2013; Marhe, Waters, et al. 2013) were excluded. In the study by Jones et al., it is stated that males were excluded due to the drug used in the smoking cessation clinical trial. In the studies by Marhe, Luijten, et al. (2013) and Marhe, Waters, et al. (2013), pregnant women were excluded due to the imaging method used in the studies.

### 3.3 Attentioanal Bias Evaluation

Two studies employed visual attention tasks (Garland et al. 2012; Waters, Shiffman, Bradley, et al. 2003), while the remaining eleven studies utilized a Stroop task. Various software programs were used for AB tasks, including E‐Prime 2.0 (Janes et al. 2010; Garland et al. 2012), PsyScope 1.2.5 PPC (Cox et al. 2002), Superlab 1.04 for Microsoft Windows 3.1 (Carpenter et al. 2006), and Experimental Laboratory software version 2 (Waters, Shiffman, Bradley, et al. 2003; Waters, Shiffman, Sayette, et al. 2003). Marhe, Waters, et al. (2013) employed an HP iPAQ Pocket PC running the Microsoft Windows Pocket PC operating system for ecological momentary assessment (EMA).

In the Stroop task, substance‐specific and neutral words were presented in different colors, and participants were instructed to respond to the color of the word as quickly as possible. In contrast, Kennedy et al. (2014) instructed participants to respond to the number of words as quickly as possible. Cox et al. used one practice block consisting of 120 stimuli; Janes et al. used four blocks of 33 trials; Marissen et al. (2006) used 100 trials (50 neutral, 50 heroin); Carpenter et al. (2006) used two blocks of 50 trials; Snelleman et al. (2015) used three blocks of 33 trials; Marhe, Waters, et al. (2013) used two blocks of 33 trials; and Waters, Shiffman, Sayette, et al. (2003) used three blocks of 32 trials. Kennedy et al. (2014) used individual‐specific drug‐related words, with each Stroop task consisting of 89 words. Marhe, Luijten, et al. (2013) used 18 blocks of 10 words each. Mitchell et al. (2013) had participants complete six trials consisting of 105 stimuli in a Stroop task. Powell et al. (2010) implemented the Stroop task using paper instead of a computer screen, with an array of 88 words for each word set.

In the study by Garland et al. (2012), a spatial cueing task was used to assess AB using alcohol‐related and neutral pictures. Alcohol‐related and neutral photographs were displayed for 200 ms, followed by the appearance of a single dot in one photograph and a double dot in the other. Participants responded using a keyboard in a procedure consisting of 160 trials. Waters, Shiffman, Bradley, et al. (2003) used 160 experimental blocks, with the fixation cross and images remaining on the screen for 500 ms.

### 3.4 Relapse Evaluation

There is no consensus on a common definition of relapse in the studies. In four studies, any level of substance use was considered a relapse (Marissen et al. 2006; Garland et al. 2012; Kennedy et al. 2014; Marhe, Waters, et al. 2013). In the study conducted by Snelleman et al. (2015), experiencing binge drinking at least once (defined as four drinks at one time for women and 5 drinks at one time for men) was considered a relapse. Janes et al. (2010) defined relapse according to the Society for Research on Nicotine and Tobacco's criteria (smoking more than once a week for 7 or more consecutive days or 2 or more consecutive weeks, followed by smoking less frequently). Urine drug testing was performed in four studies (Carpenter et al. 2006; Marhe, Luijten, et al. 2013; Mitchell et al. 2013; Kennedy et al. 2014). In the study by Garland et al. (2012), relapse was evaluated through staff observations and breathalyzer tests, as the study was conducted with patients in an addiction treatment center. Powell et al. (2010) evaluated salivary cotinine levels. Two studies assessed relapse using CO levels (Waters, Shiffman, Bradley, et al. 2003; Waters, Shiffman, Sayette, et al. 2003). In six of the studies, self‐reports from participants were used to evaluate relapse (Cox et al. 2002; Janes et al. 2010; Marissen et al. 2006; Marhe, Waters, et al. 2013; Kennedy et al. 2014; Snelleman et al. 2015). Five of the studies evaluated relapse at 3 months post‐treatment (Cox et al. 2002; Marissen et al. 2006; Marhe, Luijten, et al. 2013; Kennedy et al. 2014; Snelleman et al. 2015), and one study evaluated relapse at 6 months post‐treatment (Garland et al. 2012). Marhe, Waters, et al. (2013) evaluated early relapse on the fourth day and at the end of the PDA study week, while late relapse was assessed post‐study. Powell et al. (2010) examined relapse approximately 7 days, 30 days, and 3 months following smoking cessation. Waters, Shiffman, Bradley, et al. (2003) and Waters, Shiffman, Sayette, et al. (2003) monitored relapse through follow‐up visits up to the third month after smoking cessation. In two studies, participants were followed weekly for relapse (Janes et al. 2010; Carpenter et al. 2006). However, in two studies, the number of individuals with and without relapse was not clearly reported (Carpenter et al. 2006; Mitchell et al. 2013). Based on studies reporting the incidence of relapse, 304 participants (43%) experienced a relapse, whereas 402 participants (57%) did not.

### 3.5 Longitudinal Associations Between Attentional Bias and Relapse

While some studies examined in the current research indicate a relationship between relapse and AB related to the substance (Garland et al. 2012; Janes et al. 2010; Marissen et al. 2006; Mitchell et al. 2013; Waters, Shiffman, Sayette, et al. 2003), others suggest the opposite (Cox et al. 2002; Marhe et al. 2013a; Kennedy et al. 2014; Snelleman et al. 2015; Waters, Shiffman, Bradley, et al. 2003). Cox et al. (2002) reported no significant difference in Stroop effect scores between participants who relapsed post‐discharge and those who did not. Notably, AB related to alcohol increased in the second Stroop task among participants who relapsed, a pattern not observed in those who did not relapse. Marhe, Luijten, et al. (2013) assessed AB to cocaine using the Stroop effect. Their findings showed that the Stroop effect did not predict relapse at three months. However, the small sample size (*n* = 14) limited the generalizability of these results. Kennedy et al. (2014) found no significant difference between relapse and non‐relapse groups in cocStroop (neutral cocaine) and cStroop (incompatible‐compatible) procedure scores, citing the small sample size (*n* = 35) as a limitation. Snelleman et al. (2015) administered the Stroop procedure twice, evaluating the relationship between the Stroop effect and relapse on two occasions, and found that AB did not predict relapse in either evaluation. Marissen et al. (2006) discovered that pretreatment AB related to heroin cues predicted posttreatment relapse, though posttreatment Stroop performance did not correlate with relapse, attributed to weak reliability analysis results. The study's large sample size (*n* = 106) is noteworthy. Garland et al. (2012) found that AB related‐alcohol significantly predicted relapse (*n* = 47). Janes et al. (2010) found a relationship between the Stroop task and relapse. Carpenter et al. (2006) assessed relapse not as a categorical variable but through posttreatment positive urine rates, finding significant associations between the Stroop effect and positive urine rates only in individuals who primarily used cocaine. Mitchell et al. (2013) reported that the Stroop effect was positively correlated with the maximum number of consecutive days of reported cocaine abstinence during treatment and negatively correlated with the percentage of positive urine tests (*r* = 0.78, *p* < 0.05; *r* = −0.66, *p* < 0.05). Notably, some studies included in the review found that AB predicts relapse within the first week but not later relapse (Powell et al. 2010; Marhe, Waters, et al. 2013). Waters, Shiffman, Sayette, et al. (2003) also reported that AB predicts relapse in the first week and the time to subsequent relapse. In another study, Waters, Shiffman, Bradley, et al. (2003) used a dot probe task and found that AB did not predict the time until the first lapse.

## 4 Discussion

In the current systematic review, longitudinal studies examining the role of AB in predicting relapse have been analyzed. The reviewed studies indicate conflicting results regarding the role of AB in predicting relapse. While some studies suggest that AB predicts relapse, others do not support this relationship. Nevertheless, the findings from these studies provide significant contributions to the literature.

Evidence supporting the predictive role of AB in relapse has been presented in eight of the reviewed studies (Marhe, Waters, et al. 2013; Powell et al. 2010; Waters, Shiffman, Sayette, et al. 2003; Mitchell et al. 2013; Marissen et al. 2006; Carpenter et al. 2006; Janes et al. 2010; Garland et al. 2012). In addition, it is noteworthy that the sample sizes of these studies are larger than those of studies claiming that AB does not predict relapse. One prominent finding among the reviewed studies is the evidence showing a relationship between AB and relapse within one week (Marhe, Waters, et al. 2013; Powell et al. 2010; Waters, Shiffman, Sayette, et al. 2003). This can be explained by theories suggesting that AB is related to motivation for substance use and that AB changes alongside evolving motivational patterns during the addiction process (Field et al. 2016). Addiction treatment is a long and complex process that involves fluctuating motivations regarding substance use and abstinence. In this context, individuals' motivations for substance use vary throughout the treatment process. There are strong relationships between AB and craving. However, medical treatment approaches and psychosocial support may reduce individuals' cravings and motivations for substance use over time. Thus, it is understandable that there is a relationship between AB and recently assessed relapse.

Several possible reasons may contribute to the inconsistent results among the reviewed studies. Empirical evidence supports a strong theoretical foundation for the relationship between SUD and AB. A meta‐analysis of 21 studies conducted by MacLean et al. (2018) reported that AB toward psychoactive substances is higher in individuals with opioid use disorder compared to both healthy control groups and non‐dependent opioid users. Previous systematic reviews and meta‐analyses suggest similar results for cannabis, alcohol, and stimulants (Field et al. 2009; O'Neill et al. 2020). Substances with strong reinforcing properties can cause structural and functional changes in the brain, which may also affect relapse outcomes (Böhmer et al. 2023; Du et al. 2024). However, various factors, including medical treatments for addiction, stressful life events, coping skills, the presence of support systems, and psychiatric comorbidities, comprise complex processes that influence relapse outcomes (Del Palacio‐Gonzalez et al., 2024, Mao et al. 2024; Medenblik et al. 2024; Rahman et al. 2016). In this context, the relationships between AB and relapse may not be as clear‐cut as those between AB and levels of substance use and craving. Vafaie and Kober (2022) found that cue‐induced craving predicts relapse in their meta‐analytic studies. However, the researchers also examined studies that addressed cues without evaluating AB. In this sense, it is possible that AB does not directly predict relapse, but rather that the interaction between AB and cue‐induced craving does.

The lack of a consensus definition of relapse in the studies examined may contribute to the conflicting results. In addition, some studies (Cox et al. 2002; Marissen et al. 2006; Marhe, Waters, et al. 2013) have assessed relapse solely through self‐report, raising concerns about the accuracy of the information provided by participants. It is also possible that the different tasks used to assess the AB contribute to the conflicting results. The Stroop test has been predominantly used to measure AB, but the words used have varied across studies. This raises questions about the validity of threat and neutral stimuli for all participants. Reviewing previous studies, it is evident that Wingenfeld et al. (2006) found that, in a nonclinical sample, the personalized negative stimulus‐neutral stimulus condition resulted in a more pronounced Stroop interference compared to the general negative stimulus‐neutral condition. Kennedy et al. (2014) addressed this issue by using personalized threat stimuli. Furthermore, the number of tasks participants were exposed to in AB measurement paradigms has varied across studies, which may also contribute to different outcomes. In studies conducted by Waters, Shiffman, Bradley, et al. (2003) and Waters, Shiffman, Sayette, et al. (2003), the same sample was evaluated for the predictive ability of the Stroop and dot probe tasks regarding relapse. The researchers found that AB predicted relapse in the Stroop task but not in the dot probe procedure. They explained this by stating that “the Stroop task captures a more significant bias component, is applied closer to attempts to quit the substance, or is conducted under deprivation conditions.” No evidence has been found regarding which task is more reliable for measuring AB. However, more recent studies claim that eye‐tracking measurements of AB are advantageous compared to reaction time measurements. Reasons include the reported low reliability of reaction time measurements and the additional error variance introduced by the time taken to press a button in response to stimuli (Clauss et al. 2022). In the literature search conducted for this systematic review, no studies examining the relationship between eye‐tracking and relapse were found, and thus, eye‐tracking studies could not be included in this review.

## 5 Strengths and Limitations

This systematic review provides a comprehensive analysis of longitudinal studies examining the role of AB in predicting relapse. The review includes a wide range of studies, offering a broad perspective on the topic and contributing valuable insights into the literature. Although the results among the reviewed studies are conflicting, they still provide important information regarding the potential role of AB in relapse prediction.

The differences in the AB tasks are noteworthy when the results of these studies are evaluated. Discussions on the reliability of the dot probe task and Stroop task paradigms commonly used in AB measurement have been going on for a long time (Ataya et al. 2012; Jones et al. 2018; Xu et al. 2024). Although factors such as the ease of applying these paradigms and low cost are important, it is seen that doubts about reliability should not be ignored. In this sense, it is thought that more scientific studies to increase the reliability of the dot probe task and Stroop task are important. At the same time, attempts to determine application standards (number of blocks, stimulus presentation time, etc.) for these paradigms are suggested. In addition, it is thought that eye‐tracking studies may be useful to overcome the limitation caused by the response time in classical AB tasks.

The clinical relevance of understanding AB's potential predictive ability for relapse is noteworthy. It may help in tailoring treatment plans more effectively, where individuals with higher levels of AB could benefit from more focused interventions. In addition, AB measurements might be useful in monitoring and evaluating progress during treatment, allowing for timely adjustments to interventions.

Exploring the relationship between AB and relapse could also inform early intervention strategies, which might help in reducing relapse risk and improving recovery outcomes. Furthermore, understanding AB's role in relapse has implications for public health policies, potentially aiding in the development of more effective addiction prevention programs.

Raising awareness about the possible effects of AB on addiction and relapse could enhance community understanding and support for individuals dealing with addiction. This knowledge might also be beneficial for families and support systems, helping them provide better support to those in recovery.

One limitation of this systematic review is the exclusive focus on English‐language studies. This approach may have led to the exclusion of relevant studies published in other languages, potentially overlooking valuable data that could enrich the findings. In addition, the small sample sizes in many of the reviewed studies pose a challenge, underscoring the need for a comprehensive meta‐analysis to better understand the relationship between AB and relapse. However, conducting such a meta‐analysis is complicated by the diverse methodologies and procedures used across studies, as well as the variability in statistical outcomes and relapse definitions. These factors contribute to the difficulty in synthesizing the data into a cohesive analysis.

## 6 Conclusion

This systematic review highlights several key findings that suggest important directions for future research in the field of AB and relapse. Despite the conflicting results regarding the predictive role of AB in relapse, the relationship between AB and craving for substances, as well as the level of substance use in addiction, underscores the significance of this topic. In this context, it is suggested that further studies should be conducted on the relationship between AB and relapse, taking into account the limitations of existing research.

The findings of this study are also considered clinically significant. Informing and supporting individual's post‐addiction treatment on how to cope with AB and substance‐related issues is deemed essential for professionals working in the field of addiction. In this regard, studies examining the effects of interventions aimed at modifying AB toward substances on relapse are recommended.

Understanding the potential of AB to predict relapse may help in tailoring treatment plans more effectively. Individuals with higher levels of AB could benefit from more intensive or targeted interventions. In addition, AB measurements might be useful in monitoring and evaluating progress during treatment, allowing for timely adjustments to interventions.

Early intervention strategies could be formulated in scenarios where AB predicts relapse, which is crucial for mitigating relapse risk and enhancing recovery outcomes. Moreover, the capacity of AB to forecast relapse holds significant implications for public health policies, facilitating the creation of more efficacious addiction prevention programs.

In conclusion, the findings of this review underscore the importance of continued research into the role of AB in addiction and relapse. By addressing the identified gaps and standardizing research approaches, future studies can provide clearer insights and more effective interventions for individuals struggling with addiction.

## Author Contributions

**Zehra Su Topbaş**: conceptualization, investigation, writing–original draft, visualization, methodology, writing–review and editing, data curation, project administration. **Eda Albayrak**: conceptualization, methodology, visualization, investigation. **Nuray Şimşek**: conceptualization, investigation, project administration. **Emre Usta**: conceptualization, methodology.

## Conflicts of Interest

The authors declare no conflicts of interest.

### Peer Review

The peer review history for this article is available at https://publons.com/publon/10.1002/brb3.70300.

## Supporting information




**Table 1** Quality assessment results according to JBI Critical Appraisal Checklist for Cohort Studies

Supplementary Material 2 Search Strategy

## Data Availability

All the required information is available in the manuscript itself. The data that supports the findings of this study are available in the Supporting Information Material of this article
