# Supplementary material for: Does Attentional Bias Predict Relapse in Addiction? A Systematic Review of Longitudinal Studies
Source: Brain Behav. 2025 Feb 28;15(3):e70300. doi: 10.1002/brb3.70300 (PMC11870792; doi:10.1002/brb3.70300)
Supplement: Supplementary file 1 — Table 1 Quality assessment results according to JBI Critical Appraisal Checklist for Cohort Studies [file BRB3-15-e70300-s001.docx]

**Supplementary Material 1**

**Table 1.** Quality Assessment Results according to JBI Critical Appraisal Checklist for Cohort Studies

| **Authors** | **Quality Assessment** | | | | | | | | | | | |
| --- | --- | --- | --- | --- | --- | --- | --- | --- | --- | --- | --- | --- |
|  | **1** | **2** | **3** | **4** | **5** | **6** | **7** | **8** | **9** | **10** | **11** |  |
| Cox et al.,2002 | Y | Y | Y | U | U | U | Y | U | Y | Y | Y |  |
| Waters et al.,2003a | Y | Y | Y | N | N | U | Y | Y | N | N | Y |  |
| Waters et al.,2003b | Y | Y | Y | U | N | U | Y | Y | U | U | Y |  |
| Marissen et al.,2006 | Y | Y | Y | Y | Y | U | Y | Y | Y | N | Y |  |
| Carpenter et al.,2006 | Y | Y | Y | N | N | U | Y | Y | U | U | Y |  |
| Janes et al., 2010 | Y | Y | Y | Y | U | U | Y | Y | Y | Y | U |  |
| Powell et al.,2010 | Y | Y | Y | U | U | U | Y | Y | N | N | Y |  |
| Garland et al.,2012 | Y | Y | Y | N | N | U | Y | Y | Y | U | Y |  |
| Marhe et al.,2013a | Y | Y | Y | Y | Y | U | Y | Y | Y | U | Y |  |
| Marhe et al.,2013b | Y | Y | Y | N | N | U | Y | Y | N | N | Y |  |
| Mitchell et al.,2013 | Y | Y | Y | N | N | U | Y | Y | N | N | Y |  |
| Kennedy et all.,2014 | Y | Y | Y | N | N | U | Y | Y | U | U | Y |  |
| Snelleman et all.,2015 | Y | Y | Y | Y | Y | U | Y | Y | N | N | Y |  |

Y:Yes, U:Unclear N: No

**JBI Critical Appraisal Checklist for Cohort Studies**

1. Were the two groups similar and recruited from the same population?
2. Were the exposures measured similarly to assign people to both exposed and unexposed groups?
3. Was the exposure measured in a valid and reliable way?
4. Were confounding factors identified?
5. Were strategies to deal with confounding factors stated?
6. Were the groups/participants free of the outcome at the start of the study (or at the moment of exposure)?
7. Were the outcomes measured in a valid and reliable way?
8. Was the follow up time reported and sufficient to be long enough for outcomes to occur?
9. 9. Was follow up complete, and if not, were the reasons to loss to follow up described and explored?
10. Were strategies to address incomplete follow up utilized?
11. Was appropriate statistical analysis used?
