# Supplementary material for: Does Attentional Bias Predict Relapse in Addiction? A Systematic Review of Longitudinal Studies
Source: Brain Behav. 2025 Feb 28;15(3):e70300. doi: 10.1002/brb3.70300 (PMC11870792; doi:10.1002/brb3.70300)
Supplement: Supplementary file 2 — Supplementary Material 2 Search Strategy [file BRB3-15-e70300-s002.docx]

**Supplementary Material 2**

**Search Strategy**

| **Database** | **Search term** | **Limitation and Number of articles** |
| --- | --- | --- |
| Pubmed | ((addiction OR dependence OR substance abuse) AND (relapse)) AND (attentional bias OR Attentional blink OR Executive function OR stroop OR dot probe OR Visual probe task OR Electrophysiological signals OR Reaction time OR Cognitive bias OR Dual-task procedure OR Visual probe task OR Reward learning OR Sign-tracking OR Spatial Cueing Task)) | Clinical Trial and English  = 150 |
| Web of Science | TS=(attentional bias AND addiction AND relapse) OR TS=(attentional bias AND dependence AND relapse) OR TS=(attentional bias AND substance AND relapse) OR TS=(stroop AND dependence AND relapse) OR TS=(stroop AND addiction AND relapse) OR TS=(stroop AND substance AND relapse) OR TS=(dot probe AND dependence AND relapse) OR TS=(dot probe AND addiction AND relapse) OR TS=(dot probe AND substance AND relapse) OR TS=(Attentional blink AND substance AND relapse) OR TS=(Attentional blink AND dependence AND relapse) OR TS=(Attentional blink AND addiction AND relapse) OR TS=(Executive function AND substance AND relapse) OR TS=(Executive function AND dependence AND relapse) OR TS=( Executive function AND addiction AND relapse) OR TS=( Visual probe task AND substance AND relapse) OR TS=(Visual probe task AND dependence AND relapse) OR TS=( Visual probe task AND addiction AND relapse) OR TS=( Visual probe task AND substance AND relapse) OR TS=(Visual probe task AND dependence AND relapse) OR TS=( Visual probe task AND addiction AND relapse) OR TS=(Electrophysiological signals AND substance AND relapse) OR TS=(Electrophysiological signals AND dependence AND relapse) OR TS=(Electrophysiological signals AND addiction AND relapse) OR TS=(Reaction time AND substance AND relapse) OR TS=(Reaction time AND dependence AND relapse) OR TS=(Reaction time AND addiction AND relapse) OR TS=(Cognitive bias AND substance AND relapse) OR TS=(Cognitive bias AND dependence AND relapse) OR TS=(Cognitive bias AND addiction AND relapse) OR TS=(Dual-task procedure AND substance AND relapse) OR TS=(Dual-task procedure AND dependence AND relapse) OR TS=(Dual-task procedure AND addiction AND relapse) OR TS=( Visual probe task AND substance AND relapse) OR TS=(Visual probe task AND dependence AND relapse) OR TS=(Visual probe task AND addiction AND relapse) OR TS=(Reward learning AND addiction AND relapse) OR TS=(Sign-tracking AND addiction AND relapse) OR TS=(Spatial Cueing Task AND addiction AND relapse) | Article and English= 765 |
| EBSCO | AB (dependence OR addiction OR substance) AND AB relapse AND AB ( attentional bias OR attentional blink OR Spatial Cueing Task OR stroop OR dot probe OR Visual probe task OR Electrophysiological signals OR Reaction time OR Cognitive bias OR Dual-task OR Reward learning OR Sign-tracking ) | Academic Journals, English, Full Text= 311 |
| SCOPUS | ( ( TITLE-ABS-KEY ( addiction ) OR TITLE-ABS-KEY ( dependence ) OR TITLE-ABS-KEY ( substance ) ) ) AND ( ( TITLE-ABS-KEY ( attentional AND bias ) OR TITLE-ABS-KEY ( attentional AND blink ) OR TITLE-ABS-KEY ( executive AND function ) OR TITLE-ABS-KEY ( stroop ) OR TITLE-ABS-KEY ( dot AND probe ) OR TITLE-ABS-KEY ( visual AND probe AND task ) OR TITLE-ABS-KEY ( electrophysiological AND signals ) OR TITLE-ABS-KEY ( reaction AND time ) OR TITLE-ABS-KEY ( cognitive AND bias ) OR TITLE-ABS-KEY ( dual-task AND procedure ) OR TITLE-ABS-KEY ( visual AND probe AND task ) OR TITLE-ABS-KEY spatial AND cueing AND task ) OR TITLE-ABS-KEY (sign-tracking) OR TITLE-ABS-KEY ( reward AND learning)) ) AND ( TITLE-ABS-KEY ( relapse ) ) AND ( LIMIT-TO ( DOCTYPE , "ar" ) ) AND ( LIMIT-TO ( LANGUAGE , "English" ) ) | English = 803 |

**PUBMED**

<https://pubmed.ncbi.nlm.nih.gov/?term=%28%28addiction+OR+dependence+OR+substance+abuse%29+AND+%28relapse%29%29+AND+%28attentional+bias+OR+Attentional+blink+OR+Executive+function+OR+stroop+OR+dot+probe+OR+Visual+probe+task+OR+Electrophysiological+signals+OR+Reaction+time+OR+Cognitive+bias+OR+Dual-task+procedure+OR+Visual+probe+task+OR+Reward+learning+OR+Sign-tracking+OR+Spatial+Cueing+Task%29%29&filter=pubt.clinicaltrial&filter=lang.english>

**WOS**

<https://www.webofscience.com/wos/woscc/summary/bbc49f45-7e69-402b-a491-e4145ebd6376-0115d5e543/relevance/1>

**EBSCO**

<https://search.ebscohost.com/login.aspx?direct=true&db=asn&db=asb&db=ast&db=air&db=bpr&db=bsu&db=e5h&db=nlebk&db=eir&db=eric&db=hev&db=8gh&db=hsr&db=lxh&db=f6h&db=e864sww&db=cmedm&db=n5h&db=nsm&db=ddu&db=bwh&db=ssr&db=trh&db=obo&db=uvt&bquery=AB+(+dependence+OR+addiction+OR+substance+)+AND+AB+relapse+AND+AB+(+attentional+bias+OR+attentional+blink+OR+Spatial+Cueing+Task+OR+stroop+OR+dot+probe+OR+Visual+probe+task+OR+Electrophysiological+signals+OR+Reaction+time+OR+Cognitive+bias+OR+Dual-task+OR+Reward+learning+OR+Sign-tracking+)&cli0=FT&clv0=Y&type=1&searchMode=Standard&site=ehost-live>

**SCOPUS**

<https://www.scopus.com/results/results.uri?sort=plf-f&src=s&nlo=&nlr=&nls=&mltAll=t&sid=23be30b7425a6197da2a49f01b506807&sot=comb&sdt=b&cluster=scosubtype%2C%22ar%22%2Ct%2Bscolang%2C%22English%22%2Ct&sl=502&s=%28TITLE-ABS-KEY%28addiction%29+OR+TITLE-ABS-KEY%28dependence%29+OR+TITLE-ABS-KEY%28substance%29+AND+TITLE-ABS-KEY%28attentional+AND+bias%29+OR+TITLE-ABS-KEY%28attentional+AND+blink%29+OR+TITLE-ABS-KEY%28executive+AND+function%29+OR+TITLE-ABS-KEY%28stroop%29+OR+TITLE-ABS-KEY%28dot+AND+probe%29+OR+TITLE-ABS-KEY%28visual+AND+probe+AND+task%29+OR+TITLE-ABS-KEY%28electrophysiological+AND+signals%29+OR+TITLE-ABS-KEY%28reaction+AND+time%29+OR+TITLE-ABS-KEY%28cognitive+AND+bias%29+OR+TITLE-ABS-KEY%28dual-task+AND+procedure%29+OR+TITLE-ABS-KEY%28visual+AND+probe+AND+task%29+OR+TITLE-ABS-KEY%28Reward+learning%29+OR+TITLE-ABS-KEY%28Sign-tracking%29+OR+TITLE-ABS-KEY%28Spatial+Cueing+Task%29+AND+TITLE-ABS-KEY%28relapse%29%29&origin=searchbasic&zone=leftSideBar&editSaveSearch=&txGid=60082f52e50da6d24af729b886962030&sessionSearchId=23be30b7425a6197da2a49f01b506807&limit=10>
